# Self-rated health in adolescence as a predictor of ‘multi-illness’ in early adulthood: A prospective registry-based Norwegian HUNT study

**DOI:** 10.1016/j.ssmph.2020.100604

**Published:** 2020-05-20

**Authors:** Øystein Hetlevik, Eivind Meland, Karl Ove Hufthammer, Hans J. Breidablik, David Jahanlu, Tina L. Vie

**Affiliations:** a Department of Global Public Health and Primary Care, University of Bergen, 5020, Bergen, Norway; b Centre for Clinical Research, Haukeland University Hospital, Bergen, Norway; c Centre of Health Research, Førde Hospital Trust, PO Box1000, 6807, Førde, Norway; d Faculty of Health Sciences, Oslo Metropolitan University, PO Box 4, St. Olavs Plass, 0130, Oslo, Norway

**Keywords:** Norway, Self-rated health, Health complaints, Diagnosis, Adolescent, Early adulthood, General practice, Latent class analyses

## Abstract

Self-rated health (SRH) is a marker of future health and a possible predictor of future multimorbidity, which is a major challenge for population health and health care. There is a lack of studies on adolescent SRH and patterns of health problems across the transitional period from adolescence to early adulthood. Therefore, this study aimed to identify groups of people with similar health problems in early adulthood and explore the predictive value of adolescent SRH on the group classification after a period of 10–19 years. Data from 8828 adolescents participating in the Young HUNT-1 survey (1995–1997) were linked to the Norwegian registry of general practitioner (GP) claims, which includes diagnoses recorded in GP consultations in 2006–2014. We used latent class analysis (LCA) to identify groups of patients with similar health problems in early adulthood and explored SRH as a predictor of class membership using latent class regression, adjusting for baseline chronic disease, frequency of health care attendance, sex and age. The mean age at baseline was 16 years, and 50% of the participants were female. SRH was reported as *very good* by 28%, *good* by 61% and *not good* by 11%. We identified five groups of patient classification (*classes*): *Healthy* (35%), *Infections and general problems* (26%), *Musculoskeletal problems* (21%), *Psychological problems* (6%) and *Multi-illness* (13%). We found a gradual increase in the probability of belonging to the *Healthy* class with better SRH, and an inverse pattern for the *Psychological* and *Multi-illness* classes. This pattern remained after adjusting for baseline variables. In conclusion, there is a clear association between adolescent SRH and the risk of having multi-illness in early adulthood, seen as a proxy for later multimorbidity. This finding warrants greater attention to SRH in adolescence as a possible indicator in targeted prevention of future health problems.

## Highlights

• Adolescent self-rated health (SRH) predicted health problems 10–19 years later.
• Patients were classified into five groups with similar patterns of health problems.
• Better SRH increased the probability of adults belonging to the healthiest group.
• *Not good* SRH tripled the risk for *multi-illness* compared to *very good* SRH.
• Low SRH in adolescence is a possible predictor of multimorbidity in adulthood.

## 1 Introduction

A life-course approach is needed to prevent chronic disease (Lynch & Smith, 2005). Reliable health indicators in the young population might have great value if they could be applied to target preventive actions. Self-rated health (SRH), measuring a person's subjective perception of health, is a relevant and frequently used indicator of future health (Breidablik, Meland, & Lydersen, 2008; Manor, Matthews, & Power, 2001). Studies show that SRH can be used to predict mortality (Ganna & Ingelsson, 2015; Larsson, Hemmingsson, Allebeck, & Lundberg, 2002; Vie, Hufthammer, Meland, & Breidablik, 2019), morbidity (Manor et al., 2001; Miilunpalo, Vuori, Oja, Pasanen, & Urponen, 1997) and healthcare utilisation (Hetlevik, Vie, Meland, Breidablik, & Jahanlu, 2019; Tamayo-Fonseca et al., 2015; Vingilis, Wade, & Seeley, 2007). Previous studies have also shown that adolescent SRH predicted the utilisation of primary healthcare, medicine use and death in early adulthood (Hetlevik, Vie, Meland, Breidablik, & Jahanlu, 2019; Vie et al., 2018, 2019). Homlong et al. (2015) found a strong association between impaired SRH in adolescence (age 15–16) and use of social welfare benefits in early adulthood. These findings suggest that SRH in adolescence is a reliable indicator for both mental and physical health in the future.

Subjective health-perception probably develops mainly during childhood and adolescence (Breidablik et al., 2008; Wade & Vingilis, 1999). A four-year longitudinal study in Norway showed that SRH is a relatively stable construct among adolescents (age 13–19), but it is influenced by general well-being, health behaviour and body dissatisfaction (Breidablik, Meland, & Lydersen, 2009). Changes in SRH have also been found to be predicted by health-promoting and health-deteriorating factors in the transition between adolescence and early adulthood (Breidablik et al., 2009). Although SRH can be subject to change, Vie, Hufthammer, Holmen, Meland, and Breidablik (2014) found that only 3% of young adults showed major changes in SRH (≥2 points on a 4-point SRH scale) after 11 years. These findings are in line with the perspective that SRH is a stable measure of self-identity that can influence future health and health behaviour (Jylhä, 2009).

The theory of allostasis provides a multisystem approach for understanding how daily stress relates to health and disease (Bruce S. McEwen & Gianaros, 2011; Bruce S McEwen & Stellar, 1993). According to this theory, the organism responds to perceived anticipated demands and/or stressors by activating the autonomic nervous system, different hormonal cascades and immune reactions (Jylhä, 2009; Bruce S.; McEwen & Gianaros, 2011). These mechanisms are normal adaptation processes. However, long-standing activation can result in allostatic load (AL), an integrated measure of biological dysregulation, which in turn increases the risk of ill health and diseases (Danese & McEwen, 2012; Jylhä, 2009; Bruce S.; McEwen & Gianaros, 2011). Earlier research has demonstrated a link between SRH and AL (Cohen, Janicki-Deverts, & Doyle, 2015; Danese & McEwen, 2012; Janszky, Lekander, Blom, Georgiades, & Ahnve, 2005; Lekander, Elofsson, Neve, Hansson, & Undén, 2004). Vie et al. (Vie et al., 2014) revealed that poor SRH in adolescence predicted AL in adulthood, including changes in biomarkers representing the endocrine, metabolic and anthropometric system, consistent with the theoretical perspective of AL (Jylhä, 2009). Andreasson et al. (2019) provide experimental support for the notion that inflammation can affect SRH, and suggest that the degree of sickness induced by immune-to-brain signalling is involved in the chain of mechanisms. Also Cohen et al. (2015) linked poor SRH to poor immune function, which may be a contributing mechanism linking SRH to AL. Although the paths between SRH and AL have been found both ways, Read and Grundy (2014) found that the paths from SRH to later AL were stronger than the opposite direction, suggesting that SRH may be an even earlier indicator of health problems than AL. In sum, the model of allostatic load may be a key to understand why some individuals develop a host of complex, common diseases, while others do not (Tomasdottir et al., 2016), and based on the link between SRH and AL also a model linking SRH to patients with multiple chronic conditions, referred to as multimorbidity.

Multimorbidity is one of the main challenges facing healthcare providers (Fortin, Bravo, Hudon, Vanasse, & Lapointe, 2005; Violan et al., 2014). As allostatic load influences morbidity in an unspecific manner, with manifestations in different organ systems, multimorbidity may be a manifestation of longstanding AL. A recent Norwegian study that used the AL perspective found a strong association between poor SRH in adulthood and later multimorbidity (Tomasdottir et al., 2016). Based on the association found between SRH in adolescence and later AL (Vie et al., 2014), SRH in adolescence might predict multimorbidity problems in adulthood (Perruccio, Katz, & Losina, 2012). However, since less than 10% of the early adult population is multimorbid (Fortin, Stewart, Poitras, Almirall, & Maddocks, 2012), a combination of symptoms and diseases in young adulthood might be used to identify groups of patients with compound health problems. Belonging to a group with a high frequency of several health problems may possibly be a proxy for later multimorbidity. To our best knowledge, there are no longitudinal studies on adolescent SRH and groups of patients with similar health problems across the transitional period from adolescence to early adulthood.

In Norway, 99% of the population have a regular general practitioner (GP) in a list-patient system, where the GPs function as gatekeepers related to specialized care (Norwegian Board of Health, 2017). Annually, about 70% of the Norwegian population visit their GP, with a broad spectrum of different health issues. Therefore, analysing the data from GP contacts over a nine-year period might provide valuable insights into the occurrence of health problems – symptoms and diagnoses – in early adulthood.

The aims of this study were to identify groups of patients with similar health problems in early adulthood by data-driven methods and to explore the association between SRH in adolescence and later groups of patients in early adulthood (10–19 years later).

## 2 Methods

Our study used a prospective design to follow a cohort of adolescents in the Norwegian county of Nord-Trøndelag in 1995–1997 (Young-HUNT1). We assessed different health problems in the cohort based on the diagnoses of GP consultations in 2006–2014.

### 2.1 Study population

The Young-HUNT1 invited 10,202 students aged 13–19 years, and 8981 (88%) participated (Holmen et al., 2014). The present study excluded 153 (1.7%) of those participants because they did not respond to the SRH question, resulting in a study population of 8828.

### 2.2 Data sources

GPs in Norway send a claim to the Control and Payment of Health Reimbursement (KUHR) database for each patient contact. Each claim includes a personal ID number and the type of contact and diagnostic code(s) based on the International Classification of Primary Care (ICPC-2) (Norwegian Directorate of eHealth, 2018). We retrieved all the GP claims related to consultations in 2006–2014 (approximately 10–19 years after baseline) for our study population. The participants were in their mid-twenties to mid-thirties in the follow-up period, which is hereafter denoted as *early adulthood*.

The participants’ national identity numbers linked their data from the HUNT and KUHR. HUNT generated a new set of patient identifiers for our project, which were sent to KUHR, which linked the data and sent us the linked data without the original identity numbers.

### 2.3 Grouping of GP diagnoses

We grouped the GPs’ diagnoses for analytical purposes in the present study, mainly based on the organ chapters in ICPC-2. First we defined three groups of diagnoses across ICPC-2 chapters – an infection group (ICPC-2 codes A03, A70–78, B02, B70, D70–71, D73, F70, F72–73, H70–74, K70–71, L70, N70–73, R70–83, S09–11, S70–76, T70, U70–72, X70–74 and Y70–76), an allergy/atopy group (A92, F71, R96–97, S87–88 and S98) and an injury group (A80–82, B76, D80, F79, H78–79, L72–81, N79–81, S13–19, U80, X82 and Y80) based on the assumption that infections, allergy and injuries represent health problem randomly affecting different organ systems. All other ICPC diagnoses were classified according to ICPC-2 chapters. The most frequently used chapters – digestive (D), musculoskeletal (L), neurological (N) and psychological (P) – were classified into two subgroups, based on the ICPC-2 structure: symptoms codes (numbered 01–29) and disease-specific codes (numbered 70–99). Since there were few registrations in the chapters blood (B), eye (F), ear (H) and urinary tract (U), we combined them into one group, named BFHU.

We analysed the data for both sexes combined and, to avoid bias, excluded the gender-specific diagnoses: the W chapter (pregnancy and reproductive health), the X and Y Chapters (female and male genitalia) and cervical smear screening (ICPC-2 code A981). The consultation coded with social problems (Z Chapter) and processes or procedures (ICPC-2 codes -30 to -69) were also excluded.

### 2.4 Explanatory variables

Our main explanatory variable from the Young-HUNT1 was SRH, based on the question ‘How is your health at the moment?’, which had four possible responses – *poor*, *not so good*, *good* and *very good*. Since only 50 participants (0.6%) reported *poor* SRH, we merged them with those who reported *not so good* in the analyses, and named this combined group *not good*.

We used the following variables from the baseline data as covariates:

- Information about *health care attendance* 12 months prior to the survey was used to control for a more habitual consumption of health services. This included visiting GPs, specialists, psychologists, school health services and hospitals, and categorised as zero, one or two or more (≥2) healthcare attendances.
- The variable *chronic disease* (no/yes) was used to adjust for any self-reported illness lasting ≥ 3 months and/or diabetes, epilepsy or migraine diagnosed by a physician.
- The other covariates were age and sex.

### 2.5 Outcome measurers

The outcome measure was the (absolute or relative) probability/odds of inclusion in the various (latent) classes/groups of patients with similar health problems.

### 2.6 Statistical methods

We used latent class analysis (LCA) to identify groups, called *classes* in LCA terminology, of patients with similar patterns of symptoms and diagnoses in the GP consultations. In LCA, we fit latent class models to the data, basically a type of finite mixture models which assumes that the patients can be classified into a number of discrete *classes* (the latent, *non-observed* variables). For patients *within* a class, the event of having had a specific type of diagnosis (the *observed* variables) from GP consultations is assumed to be statistically independent of the event of having had a different type of diagnosis. The model is a simplification of reality, and the assumption of independence will typically not be completely fulfilled (i.e. there will always be some remaining dependence within each class). However, the model can be used as an approximation of reality, in which patients with similar patterns of diagnoses tend to get classified into the same classes. While all types of health problems are presented in each class, the *proportion*s of patients with the various health problems differ between classes.

The observed variables in these types of models must be categorical/nominal. In our analyses, we defined them as binary variables: 1 if the patient had ≥2 GP consultations of the specified type, and 0 otherwise. We also tried fitting models with different cut-points, with similar results.

The *number* of latent classes used in the models must be fixed before fitting the models. If there are too few classes, the models will not fit the data well. On the other hand, if there are too many classes, there will be over-fitting and technical problems in actually estimating the model parameters (nonconvergence), and the resulting models will be hard to interpret. We therefore fitted the models several times, with different numbers (1–7) of classes. We used two criteria to select the number of classes in our final model: (1) the classes found by the LCA models needed to have a natural, meaningful interpretation, and (2) the interpretable model with the lowest value of the ‘Bayesian information criterion’ (BIC) was preferred

Note that the latent classes are never observed, and only the *probability* of belonging to a given class can be established for each patient. Nevertheless, the model allowed us to calculate this probability as a function of other explanatory baseline variables. We explored SRH as a predictor for class membership using latent class regression and report the odds ratios (ORs) and 95% confidence intervals (CIs) for each latent class membership. We fitted a model with SRH as the only predictor in the *unadjusted* analyses. We used the additional baseline predictors age, sex, chronic diseases and healthcare attendance in the *fully adjusted* analyses. The *very good* SRH level was used as the reference level, and the class with the least amount of reported health problems was used as the reference class. Thus, the odds ratios measure the odds of belonging to the various classes compared to the odds of belonging to the *‘Healthy’* reference class.

The LCA analyses were performed using version 1.4.1 of the ‘poLCA’ package (Linzer & Lewis, 2011) on R version 3.5.1 (R: A language and environment for statistical computing, 2018). Each model fit was repeated at least 200 times to ensure that the model fitting converged to optimal parameter estimates. The other analyses were performed using R and/or Stata version 15 (Stata Corp LLC, Texas, USA).

### 2.7 Ethics

Participation in the Young-HUNT1 study was based on written consent, and the participants have later been informed about their right to withdraw their consent. The Regional Committee of Ethics (REK sør-øst 2012/920) and the register owners approved the use and linkage of data in this study.

## 3 Results

Among the 8828 participants in the current study, 50% were female (Table 1). The participants’ mean age was 16 years (SD = 1.8 years) at baseline, and 97.1% had one or more GP consultations (with the ICPC-2 codes used in this study) during the nine-year study period. The mean number of consultations was 18.3 (SD = 18.6): 22.1 (SD = 21.0) for women and 14.5 (SD = 15.1) for men

**Table 1:** Baseline data from Young-HUNT1 (1995–1997) and symptoms and diseases in early adulthood (2006–2014) registered in GP consultations (*N* = 8828).

	Patients	Proportion/(SD)
*Baseline data Young-HUNT*		
Sex		
Female	4387	50%
Male	4441	50%
Age at survey (mean, SD)	16.0	(1.8)
Self-rated health		
Very good	2508	28%
Good	5352	61%
Not good	968	11%
Health care attendance last year		
0	3601	41%
1	3565	40%
2+	1662	19%
Chronic diseases		
No	7855	89%
Yes	973	11%

*Health problems*^a^*in follow-up period 2006–2014*		
Infections	5443	62%
Atopy	1296	15%
Injuries	1836	21%
A – general and unspecified, whole chapter	2917	33%
D – digestive symptoms (D01-29)	1811	21%
D – digestive diagnoses (D70-99)	664	8%
K – circulatory, whole chapter	658	7%
L – musculoskeletal symptoms (L01-29)	3698	42%
L – musculoskeletal diagnoses (L70-99)	2368	27%
N – neurological symptoms (N01-29)	1068	12%
N – neurological diagnoses (N70-99)	541	6%
P – psychological symptoms (P01-29)	2044	23%
P – psychological diagnoses (P70-99)	1404	16%
R – respiratory, whole chapter	1457	17%
S – skin, whole chapter	2199	25%
T – endocrine, metabolic and nutritional, whole chapter	755	9%
Others (BFHU)^b^	1357	15%
No health problems^a^ in any of the above diagnosis groups	965	11%

a ‘Health problems’ was defined as at least 2 consultations with a diagnosis from this group/chapter. Grouping: First, infections, atopy/allergy and injuries across all ICPC-2 chapters, then grouping related to chapters or part of chapters.

b BFHU = Chapters B (blood and blood forming organs) + F (eye) + H (ear) + U (urinary tract).

Table 1 gives an overview of the frequency of different symptoms and diseases identified in GP consultations, grouped by infections, atopic conditions and injuries *across all* ICPC-2 chapters, with other consultations grouped according to ICPC-2 chapters.

We found the highest frequencies of all groups of health problems among those with *not good* SRH. Also, most groups of health problems were more frequent among those with *good* SRH compared to those with *very good* adolescent SRH (results not shown).

### 3.1 Latent class analyses

**Fig. 1:**
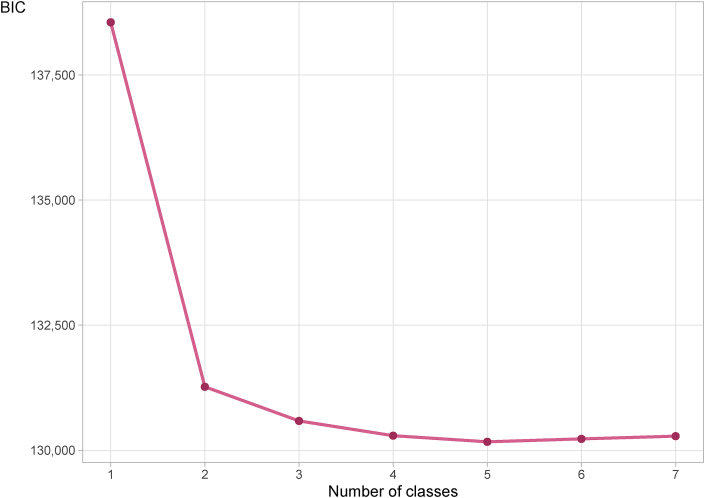
The Bayesian information criterion (BIC) for model selection in the latent class analysis for models with 1–7 classes (N = 8828). A model with 5 classes had the lowest (most optimal) BIC value.

The statistical analyses (lowest BIC) supported a final model with five classes, which represented a clinical meaningful interpretation of the classes Fig. 1. The classes used in the following analyses were named.

• *Healthy* class (35%)
• *Infections and general problems* class (26%)
• *Musculoskeletal problems* class (21%)
• *Psychological problems* class (6%)
• *Multi-illness* class (13%)

### 3.2 Classes of health problems

**Fig. 2:**
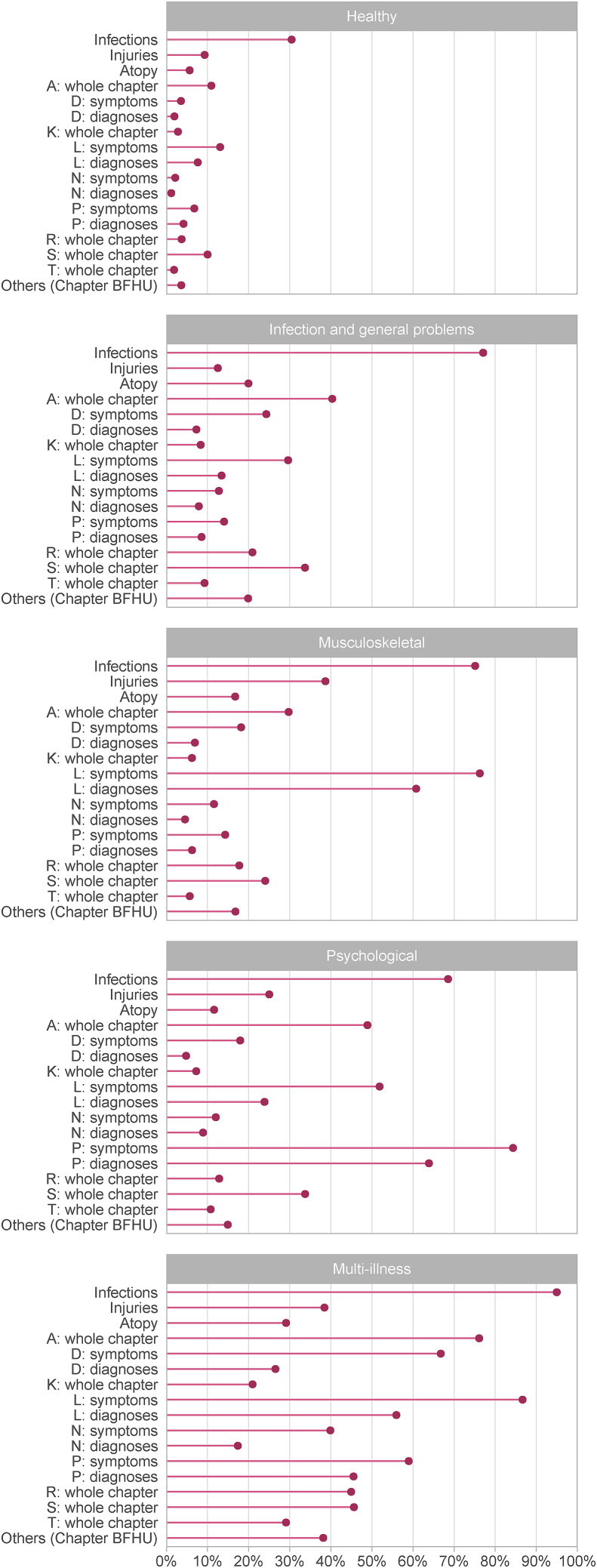
Frequency of health problems in different classes of ‘health problems’ in early adulthood (N = 8828).

The frequencies of different health problems found in the identified five classes are shown in Fig. 2.

The *Healthy* class encompassed 35% of the study population. Almost one third of the members in this class had ≥2 consultations for infections, and approximately one in ten had consultations for general problems (Chapter A), musculoskeletal (Chapter L) and/or skin-related problems (Chapter S). The patterns of symptoms and diseases in the next class, *Infections and general problems* (26%), were rather similar to the *Healthy* class, with the most frequent consultations being for infections, general, musculoskeletal and skin-related problems. However, the overall *frequency* of health problems was higher in this class than in the *Healthy* class. More than half of the members in the *Musculoskeletal* class (21%) had symptoms and/or disease diagnoses from Chapter L. In the *Psychological* class (6%), the symptoms and diagnoses from the psychological ICPC-2 chapter were predominant, together with infections. The *Multi-illness* class encompassed 13% of the population and had generally higher frequencies for most of the diagnostic groups, compared to the other classes. Psychological problems were also frequent in this class, with some 59% of the patients having ≥2 consultations for psychological symptoms, and 46% having consultations for a disease diagnosis from Chapter P.

**Fig. 3:**
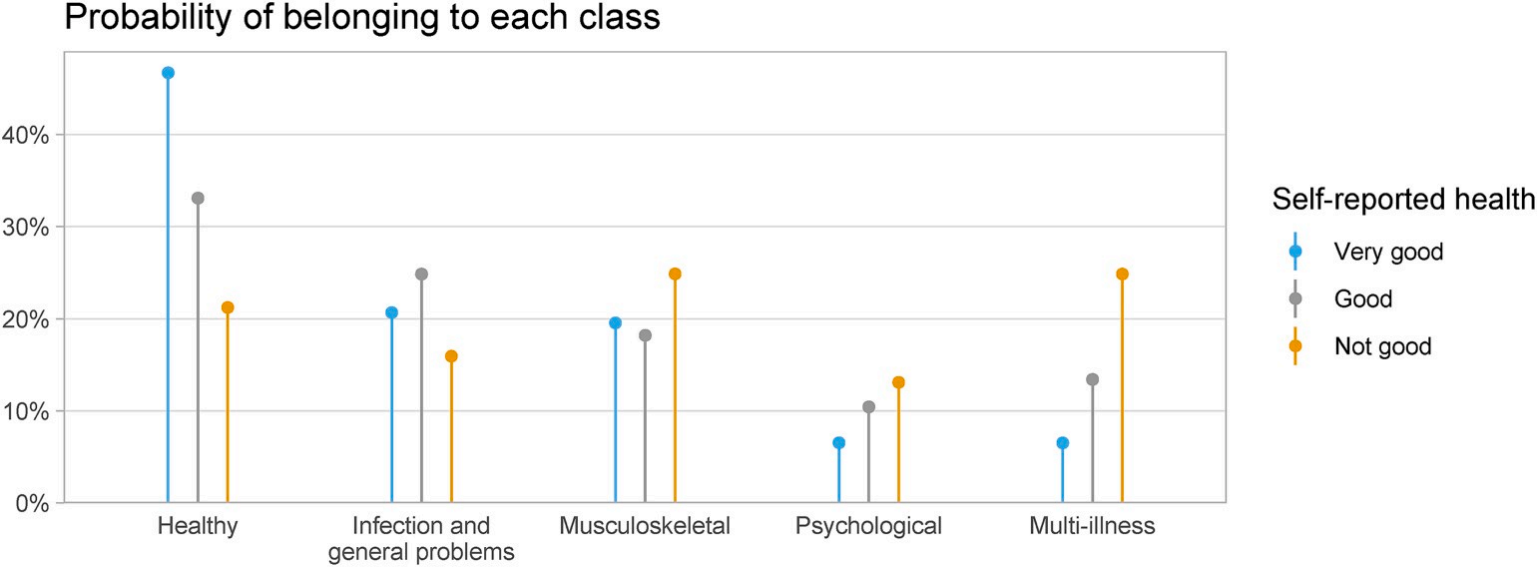
Probability of membership in different classes of ‘health problems’ in early adulthood based on self-rated health (SRH) in adolescence (N = 8828).

Fig. 3 shows the estimated probability of belonging to each of these five classes depending of the levels of SRH in adolescence.

The probability of being in the *Healthy* class in early adulthood steadily decreased with lower levels of SRH in adolescence. Among those reporting their SRH as *very good*, almost half (47%) were included in the *Healthy* class. This decreased to one third (33%) for *good* SRH and to one fifth (21%) for *not good* SRH.

While the *Infections and general problems* and *Musculoskeletal* classes had non-monotonic patterns of association with SRH, the *Psychological* class and, especially, the *Multi-illness* class had a steadily increasing probability of membership with lower SRH in adolescence.

Using adolescent SRH as a predictor of class membership Table 2, we found all the participants who did not report their SRH as *very good* had significantly higher odds of membership in all the non-*Healthy* classes, compared to the *Healthy* reference class. However, the estimated odds ratios for membership in the *Infections and general problems* class did not differ significantly between the *good* and *not good* SRH groups.

**Table 2:** Odds ratios for ‘health problems’ latent class membership in early adulthood, based on latent class regression models (*N* = 8828). The explanatory variables were self-rated health (SRH) and other baseline variables in adolescence. The *Healthy* class is used as the reference class.

	Healthy (ref.)	Infection and general problems			Musculoskeletal			Psychological			Multi-illness		
Unadjusted Model	OR	OR	95% CI	*P*-value	OR	95% CI	*P*-value	OR	95% CI	*P*-value	OR	95% CI	*P*-value
*SRH*													
Very good (ref.)	–	1.00	–	–	1.00	–	–	1.00	–	–	1.00	–	–
Good	–	1.70	1.38 to 2.09	<0.001	1.31	1.09 to 1.59	0.005	2.25	1.70 to 2.98	<0.001	2.90	2.30 to 3.66	<0.001
Not good	–	1.70	1.12 to 2.56	0.01	2.80	2.08 to 3.77	<0.001	4.41	2.95 to 6.59	<0.001	8.39	6.19 to 11.37	<0.001

**Adjusted Model**													
*SRH*													
Very good (ref.)	–	1.00	–	–	1.00	–	–	1.00	–	–	1.00	–	–
Good	–	1.60	1.26 to 2.01	<0.001	1.22	1.02 to 1.47	0.03	2.03	1.47 to 2.82	<0.001	2.42	1.90 to 3.09	<0.001
Not good	–	1.88	1.25 to 2.82	0.002	2.20	1.61 to 3.01	<0.001	3.94	2.50 to 6.22	<0.001	6.45	4.60 to 9.04	<0.001
*Age (years)*	–	0.99	0.94 to 1.05	0.79	0.98	0.93 to 1.02	0.32	0.93	0.87 to 1.01	0.07	0.92	0.87 to 0.97	0.002
*Sex*													
Female (ref.)	–	1.00	–	–	1.00	–	–	1.00	–	–	1.00	–	–
Male	–	0.06	0.04 to 0.10	<0.001	3.05	2.07 to 4.49	<0.001	0.87	0.63 to 1.20	0.39	0.16	0.13 to 0.21	<0.001
*Health care attendance*													
none	–	1.00	–	–	1.00	–	–	1.00	–	–	1.00	–	–
once	–	1.49	1.19 to 1.86	<0.001	1.11	0.92 to 1.34	0.26	1.18	0.87 to 1.59	0.28	1.40	1.13 to 1.73	0.002
Two or more times	–	1.70	1.26 to 2.29	<0.001	1.40	1.09 to 1.80	0.008	1.77	1.24 to 2.52	0.002	2.35	1.81 to 3.04	<0.001
*Chronic disease*	–	1.96	1.41 to 2.73	<0.001	1.38	1.00 to 1.91	0.05	1.76	1.16 to 2.66	0.008	2.34	1.76 to 3.13	<0.001

The ORs for SRH were somewhat attenuated in the multivariate model, which adjusted for sex, age and the baseline variables healthcare attendances and chronic disease, compared to the unadjusted model. This was most marked for the *Multi-illness* class, where the OR for *not good* SRH was reduced from 8.39 (CI: 6.19–11.37) to 6.45 (CI: 4.60–9.04). For the *not good* SRH, the OR was 3.94 (CI: 2.50–6.22) for belonging to the *Psychological* class and 2.20 (1.61–3.01) for the *Musculoskeletal* class, using *very good* SRH as the reference.

Males were over-represented in the *Healthy* class, and the sex effects observed in the other classes were related to the high proportion of males in the reference group.

Higher frequencies of healthcare attendance and reporting a chronic disease at baseline were associated with higher odds of membership in all the non-*Healthy* classes.

## 4 Discussion

### 4.1 Main findings

To the best of our knowledge, this is the first study to explore adolescent SRH as a predictor of groups of patients with similar health problems in early adulthood. Using the GPs’ diagnoses as an indicator for health problems, we showed a strong association between SRH in adolescence and different classes of patients with similar health problems in early adulthood. We found a gradual decrease in the probability of being a member of the *Healthy* class with lower levels of SRH, from 0.47 for *very good* SRH to 0.21 for *not good* SRH. About 60% of the study population attained membership in the *Healthy* or *Infections and general problems* classes. The pattern of problems is similar in these two classes, although the frequency of problems is higher in the latter class. However, females were markedly at risk for membership in the *Infections and general problems* class, probably because they had a generally higher frequency of care contact, even when reproductive reasons were excluded (Wang, Hunt, Nazareth, Freemantle, & Petersen, 2013). These two largest classes probably both represent a rather healthy segment of the population.

In the *Psychological* class, 84% of the members had two or more consultations with psychological symptoms, and 64% had a disease diagnosis. Musculoskeletal symptoms were also frequent in this class, but disease diagnoses from the same chapter were markedly less frequent. This indicates a possible connection between musculoskeletal symptoms and psychological distress, which is in line with earlier studies (Auvinen et al., 2017; Bair, Wu, Damush, Sutherland, & Kroenke, 2008), and there is an increased probability for membership in this class with lower SRH.

Nearly all patients (95%) in the *Multi-illness* class had two or more consultations for infectious conditions, which is a common health problem in early adulthood. Nevertheless, a large majority also had problems related to ICPC-2 Chapter A, i.e. general and unspecified problems, and also digestive symptoms, musculoskeletal symptoms or psychological problems.

We have not identified studies that are comparable in this age group, but cross-sectional studies among adults confirm a clear association between SRH and multimorbidity (Mavaddat, Valderas, van der Linde, Khaw, & Kinmonth, 2014; Perruccio et al., 2012; Tomasdottir et al., 2016). The marked increase in the probability of membership in the *Multi-illness* class from *very good* through *good* to *not good* SRH indicate that worse SRH in adolescence predicts ‘membership’ in a class which has a relatively high risk of *several* diagnostic groups in early adulthood. This new knowledge supports the use of SRH as an indicator for later multimorbidity.

The *Multi-illness* class seems to represent persons with health problems related to many bodily systems that could be related to high AL, an association shown in a review paper (Beckie, 2012). The pronounced associations between low adolescent SRH and multi-illness in early adulthood could also support the theory linking SRH to AL (Jylhä, 2009; Vie et al., 2014). Numerous studies on neuro-immuno-physiology document how subjective health and health complaints are associated with basic physiological processes (Beckie, 2012; Jylhä, 2009). SRH also seems to be a general subjective measure of a person's wellbeing, in some way interpreting the physiological impulses related to AL. According to the theory of allostasis, such impulses can be present before long-standing stress activation results in more pronounced symptoms or diseases (Beckie, 2012; Jylhä, 2009; Bruce S.; McEwen & Gianaros, 2011).

Although SRH is a rather stable construct, Bauldry, Shanahan, Boardman, Miech, and Macmillan (2012) found that SRH, from childhood to adulthood, was affected by the young person's health behaviour. They concluded that health promotion during adolescence, as a strategically important period for establishing future health behaviour, might improve adult health. Intervention studies aiming to improve subjective wellbeing among adolescents in school settings, as well as web-based interventions, mostly applying cognitive behaviour therapy in clinical and high-risk young populations, are also promising. A Swedish study showed that a school-based cognitive behavioural program for depression prevention positively influenced SRH over 12 months (Garmy, Clausson, Berg, Steen Carlsson, & Jakobsson, 2019). These studies indicate that preventive measures can improve SRH in adolescence. However, we don't have evidence of a long-term effect. The association between poor SRH and AL could be circumstantial evidence that improving SRH positively influences future health. As such, SRH may be a suitable measure for public health interventions, which would target preventive actions at the group level for persons with poor SRH. Clinicians may use SRH as a marker for various subclinical conditions, prevention and/or early morbidity. However, there is still a lack of evidence for the usefulness of interventions based on adolescents' SRH. Further research on interventions is needed before such strategies can be recommended.

### 4.2 Strengths and limitations

This study's strengths include the high response rate (88%) in the Young-HUNT1 survey, the long follow-up period and the long period for registration of GP diagnoses. Norway has universal healthcare coverage with a list-patient system for GP services that covers the total population. Since all GP consultations are registered in the KUHR database, selection bias related to GPs' interest in participating in research is eliminated. Other strengths include using registries, which avoids recall bias, and the study's longitudinal cohort design.

A limitation of the study is that mostly one and rarely more diagnoses were noted for each consultation. Therefore, we might underestimate the health problems presented to GPs, since GPs typically deal with 3–4 health issues during each consultation (Bjørland & Brekke, 2015). However, with a follow-up period of nine years, we assume that long-standing health problems have been captured by the GPs’ diagnoses. A cut-off point of 2 consultations within a group of diagnoses was set to account for multi-illness and to reduce the number of random cases with a possible misdiagnosis.

There are variations among individuals regarding when they seek healthcare for symptoms or problems, and our method (i.e. using GP reports) do not give a complete overview of health problems in the study population. However, using data from a nine-year period increases the probability of catching a broad spectrum of health problems. Nevertheless, GP reports may reflect different patterns of healthcare attendance already established at baseline. Such bias is partly compensated for by the variable ‘frequency of health care attendances’ that was measured at baseline.

Another limitation of the present study is the lack of information on socioeconomic status, which is an important predictor of overall health and SRH (Ambresin, Chondros, Dowrick, Herrman, & Gunn, 2014; Goodman, Huang, Schafer-Kalkhoff, & Adler, 2007).

## 4.3 Conclusion

While most individuals have good health in early adulthood, some groups of patients have a noticeable burden of health problems. There is a clear association between the adolescent SRH and the risk of having a multi-illness in early adulthood, seen as a proxy for later multimorbidity, with participants who report *not good* SRH having an especially higher risk. This warrants great attention to SRH in adolescence as an indicator of future health problems and a help to introduce targeted preventive measures. However, the current knowledge on convenient interventions is scarce. It is, therefore, important to expand knowledge in this field to further understand the specific predictive power of SRH, as well as factors influencing SRH.

## Ethical approvals concerning the paper

Participation in the Young-HUNT1 study was based on written consent, and the participants have later been informed about their right to withdraw their consent.

The Regional Committee of Ethics (REK sør-øst 2012/920) and the register owners - The Norwegian Directorate of Health and HUNT Research Centre at the Faculty of Medicine and Health Sciences, Norwegian University of Science and Technology NTNU - approved the use and linkage of data in this study.

## CRediT authorship contribution statement

**Øystein Hetlevik:** Conceptualization, Methodology, Writing - original draft. **Eivind Meland:** Conceptualization, Methodology, Writing - review & editing. **Karl Ove Hufthammer:** Conceptualization, Methodology, Formal analysis, Writing - review & editing. **Hans J. Breidablik:** Conceptualization, Writing - review & editing. **David Jahanlu:** Conceptualization, Writing - review & editing. **Tina L. Vie:** Conceptualization, Methodology, Writing - review & editing.

## Declaration of competing interest

None declared by all the authors.
